# Physical and Antibacterial Properties of Peppermint Essential Oil Loaded Poly (*ε*-caprolactone) (PCL) Electrospun Fiber Mats for Wound Healing

**DOI:** 10.3389/fbioe.2019.00346

**Published:** 2019-11-26

**Authors:** Irem Unalan, Benedikt Slavik, Andrea Buettner, Wolfgang H. Goldmann, Gerhard Frank, Aldo R. Boccaccini

**Affiliations:** ^1^Department of Materials Science and Engineering, Institute of Biomaterials, Friedrich-Alexander-University Erlangen-Nuremberg, Erlangen, Germany; ^2^Chair of Aroma and Smell Research, Department of Chemistry and Pharmacy, Friedrich-Alexander-University Erlangen-Nuremberg, Erlangen, Germany; ^3^Department of Physics, Institute of Biophysics, Friedrich-Alexander-University Erlangen-Nuremberg, Erlangen, Germany

**Keywords:** peppermint essential oil, poly (ε-caprolactone), electrospinning, antibacterial activity, wound healing

## Abstract

The aim of this study was to fabricate and characterize various concentrations of peppermint essential oil (PEP) loaded on poly(ε-caprolactone) (PCL) electrospun fiber mats for healing applications, where PEP was intended to impart antibacterial activity to the fibers. SEM images illustrated that the morphology of all electrospun fiber mats was smooth, uniform, and bead-free. The average fiber diameter was reduced by the addition of PEP from 1.6 ± 0.1 to 1.0 ± 0.2 μm. Functional groups of the fibers were determined by Raman spectroscopy. Gas chromatography-mass spectroscopy (GC-MS) analysis demonstrated the actual PEP content in the samples. *In vitro* degradation was determined by measuring weight loss and their morphology change, showing that the electrospun fibers slightly degraded by the addition of PEP. The wettability of PCL and PEP loaded electrospun fiber mats was measured by determining contact angle and it was shown that wettability increased with the incorporation of PEP. The antimicrobial activity results revealed that PEP loaded PCL electrospun fiber mats exhibited inhibition against *Staphylococcus aureus* (gram-positive) and *Escherichia coli* (gram-negative) bacteria. In addition, an *in-vitro* cell viability assay using normal human dermal fibroblast (NHDF) cells revealed improved cell viability on PCL, PCLPEP1.5, PCLPEP3, and PCLGEL6 electrospun fiber mats compared to the control (CNT) after 48 h cell culture. Our findings showed for the first time PEP loaded PCL electrospun fiber mats with antibiotic-free antibacterial activity as promising candidates for wound healing applications.

## Introduction

Human skin, the largest organ in the integumentary system, provides a robust barrier to protect the body against physical, chemical, thermal, surgical, and bacterial damages (Priya et al., 2008). The injury of human skin occurs by acute and chronic wound effects such as trauma, burns, ulcers, or diabetes. Wound dressings play a crucial role in treating these injuries by protecting the wound from the external environment, providing suitable moisture and facilitating the wound healing process (MacNeil, 2008). Further, antibacterial wound dressings are required to reduce the risk of bacterial infection during the wound healing process. Many reports have been published on the antibacterial effects of wound dressings containing antibiotics (Howell-Jones et al., 2005) and metal ions such as silver, zinc, and copper (Juby et al., 2012; Mahltig et al., 2013; Kęziora et al., 2018). Moreover, in recent years the combination of synthetic biomaterials and biological molecules based on plant-derived compounds has started to be used widely for wound healing approaches (Ramos-e-Silva and Ribeiro de Castro, 2002; Dhivya et al., 2015). Although the use of phenols and essential oils (EOs) as natural active agents is rather novel in combination with engineered biomaterials for biomedical applications (Sadri et al., 2015; Li et al., 2018; Jaganathan et al., 2019), there is increasing interest for this kind of substances in medicine, considering the fact that EOs have been used by humans for thousands of years to obtain therapeutic effects (Silva and Fernandes Júnior, 2010).

EOs based on plant extracts, such as peppermint, cinnamon, lemon, and clove essential oil, have been applied to accelerate the wound healing process since ancient times due to their various therapeutic benefits like antibacterial activity, inflammatory, and antioxidation potential (Prabuseenivasan et al., 2006; Bakkali et al., 2008). However, the volatility, low stability, and high sensitivity to environmental factors have limited the usage of EOs in wound healing applications (El Asbahani et al., 2015). In the last decade, however, research has focused on EOs-biomaterial incorporation to exploit the therapeutic properties of EOs (Bilia et al., 2014; Rijo et al., 2014; Zhang et al., 2017). One commonly considered EO is peppermint (PEP) essential oil. PEP is mostly derived from the leaf of the *Mentha piperita* (Lamiaceae) and it is composed of L-menthol, menthone, methyl acetate, and limonene, which exhibit antibacterial activity (McKay and Blumberg, 2006; Kligler and Chaudhary, 2007).

Electrospinning is a cost-effective, versatile, and well-established method to produce flexible, highly porous, nano- or micro-size, and continuous fibrous structures (Sill and von Recum, 2008). Keeping the stability of chemical structures and enhancing biological properties by incorporation of synthetic and natural polymers are further advantages of the electrospinning method (Martins et al., 2008). Various biodegradable and biocompatible polymeric materials such as poly(ε-caprolactone) (PCL), poly (L-lactic acid) (PLLA), poly (lactic-co-glycolic acid) (PLGA), collagen, chitosan, gelatin, and their copolymers have been investigated for fabricating fibrous materials (Sell et al., 2010; Mogoşanu and Grumezescu, 2014; Sun et al., 2016). Among these, PCL is the most commonly used biocompatible, low-cost, biodegradable, linear-aliphatic polyester for fiber electrospinning fabrication (Abedalwafa et al., 2013). It can be easily processed in combination with natural polymers and biomolecules to enhance antibacterial and biological properties. Also, PCL exhibits relatively high mechanical properties (Croisier et al., 2012). The development of PCL fibers by electrospinning using “benign” (non-toxic) solutions has been also investigated (Liverani and Boccaccini, 2016). In recent years, combinations of EOs and PCL have been investigated as wound dressing materials in several studies. For instance, Suganya et al. (2011) fabricated PCL and polyvinyl pyrrolidone (PVP) nanofiber mats containing herbal drug from *Tecomella undulata* by electrospinning technique. Their work revealed that incorporation with EO led to strong antibacterial properties against several bacteria strains, such as *P. aureuginosa, S. aureus*, and *E. coli*. According to Shao et al. (2011), green tea phenols (GTP)-loaded poly(ε-caprolactone)/multi-walled carbon nanotubes (PCL/MWCNTs) composite nanofibers displayed low cytotoxicity for osteoblast cells whereas high inhibition effect was observed for Hep G2 tumor cells. Jin et al. (2013) reported that human dermal fibroblasts (NHDF) proliferation was increased during 9 days of cell culture with the addition of four different plant extracts separately. In another study, Karami et al. (2013) produced thymol loaded 50/50 PCL/poly(lactic acid) (PLA) hybrid nanofibrous mats for wound healing. *In vivo* rat wound healing results indicated that the wound-closure percentage was 92.5% after 14 days compared to 68% for gauze bandages as a control. More recently, Amiri and Rahimi (2019) developed PCL electrospun nanofibers containing cinnamon oil nanoparticles, and their results revealed the positive effect of controlled cinnamon oil release in drug delivery and tissue engineering applications.

The aim of the present study was to fabricate various concentrations of peppermint oil loaded PCL electrospun fiber mats intended for antibiotic-free wound healing applications and to characterize their morphology, physical properties, and antibacterial activity. In this sense, we investigated the effect of various PEP concentrations (1.5, 3, and 6% v/v) on morphology, average fiber diameter, contact angle (wettability), *in vitro* degradation, antibacterial activity, and cell viability of PCLPEP electrospun fiber mats. The combination of PEP and PCL in fibrous structures for biomedical applications has not been investigated before, to the best of the authors' knowledge.

## Materials and Methods

### Materials

PCL (Mw = 80,000), PEP essential oil (purified by triple-distillation), standard menthol, and fetal bovine serum (FBS) were purchased from Sigma-Aldrich (Darmstadt, Germany). Glacial acetic acid (GAA, VWR, Darmstadt, Germany) was used as a solvent. Phosphate-buffered saline (PBS, biotech grade, pH 7.4) and dichloromethane (DCM) were obtained from VWR (Darmstadt, Germany). The microorganism strains of *S. aureus* (ATCC25923), and *E. coli* (ATCC25922) were used in our laboratory. Luria-Bertani (LB) agar and lysogeny broth (LB) medium were supplied by Carl Roth GmbH (Karlsruhe, Germany). Dulbecco's modified Eagle‘s medium (DMEM), penicillin/streptomycin (PS), and trypsin/EDTA were purchased from Thermo Scientific (Schwerte, Germany). NHDF cell line was obtained from Translation Research Center (TRC), Erlangen. All reagents and solvents were of analytical grade.

### Methods

#### Preparation of Electrospinning Solutions

**Figure 1:**
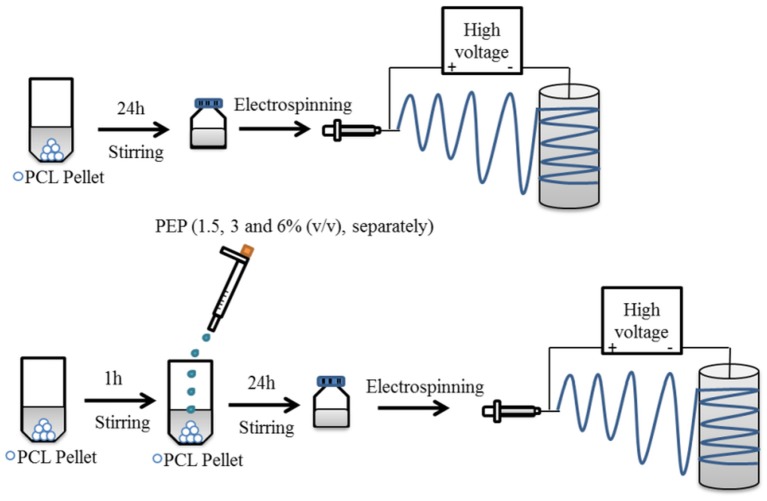
Schematic illustration for the preparation of electrospun fiber mats with and without PEP.

The preparation of electrospinning solutions is illustrated in Figure 1. PCL solution (20 w/v%) was dissolved in GAA overnight under constant stirring at room temperature to fabricate pure PCL electrospun fiber mats. For the PEP loaded PCL solutions, various concentrations of PEP [at 1.5, 3, and 6% (v/v), namely PCLPEP1.5, PCLPEP3, and PCLPEP6] were added separately into the polymer solution. The prepared solutions were stirred overnight at room temperature to yield a homogenous solution. The polymer and PEP concentrations were adjusted to the optimum electrospinning parameters.

#### Electrospinning Process

Electrospinning was carried out under defined and constant ambient conditions [temperature (T): 25°C and relative humidity (RH): 40%] by using a commercially available setup (EC-CLI, IME Technologies, Netherlands). Each electrospinning solution was loaded separately into a 3 ml plastic syringe equipped with a 23G needle. The solutions were fed at 0.4 ml/h. An aluminum sheet wrapped around a rotating drum, used as the collector, was placed at a distance of 11 cm from the needle tip to the collector. Electrospun fiber mats were collected by applying a voltage at +14 kV in the needle, and at −1 kV in the target. Further, the gas shield accessory with nitrogen flux was set at 8 ml/min for optimization of the Taylor cone. The obtained samples were stored at 4°C in the dark until further analysis.

#### Characterizations

##### Surface morphology and average fiber diameter

The morphology of the electrospun fiber mats was characterized using a scanning electron microscope [SEM, ETH: 2 kV, Everhart-Thornley detector (SE2), AURIGA base 55, Carl Zeiss]. The samples were coated with a thin layer of gold (Q150T Turbo-Pumped Sputter Coater/Carbon Coater, Quorum Technologies) before SEM observations. The average diameter and diameter distribution of electrospun fibers was estimated from the SEM micrographs via Image J analysis software (NIH, USA). The average fiber diameter was measured randomly at 50 different points for each sample.

##### Raman spectroscopy analysis

Raman spectra of the electrospun fiber mats and peppermint oil were analyzed using a μRaman spectrometer (LabRAM 800, HORIBA, Jobin Yvon) operating with a He-Ne laser source with an excitation wavelength of 633 nm. A microscope objective 50x/0.75 and a 1.800 line/mm spectrometer grating was used to record the spectra.

##### Wettability

The wettability of the electrospun fiber mats was measured by using a contact angle measuring device (DSA30 CA Measurement setup, Kruess GmbH) by the sessile drop method. The sample was pasted on a glass slide before the test, and an 8 μl droplet of de-ionized water was pipetted onto the surface of fiber mats for each measurement. Three measurements were taken at different locations of the same mat, and the average value was obtained.

##### *In vitro* degradation

*In vitro* degradation experiments were carried out as described previously (Agnes Mary and Giri Dev, 2015). Briefly, the electrospun fiber mats were cut into 3 × 3 cm^2^ pieces, which had an initial weight of around 30 mg. The samples were immersed in 10 ml of phosphate-buffered saline (PBS, pH 7.4) solution for 1, 3, 7, and 14 days at 37°C and 120 rpm. At each time interval, samples were taken out from the PBS and rinsed three times with de-ionized water prior to drying at 37°C for 3 days. The morphology of samples was analyzed by SEM. The weight loss of electrospun fiber mats was calculated as follows (1):

(1) $$\begin{array}{l} Weight\;loss\;\left(\%\right)=\frac{\left(Wi-Wd\right)}{Wi\;}x100 \end{array}$$

where wi and w_d_ are the initial and dry weight of mats, respectively. All degradation tests were carried out in triplicate.

##### Peppermint oil content in PCL electrospun fiber mats

The total PEP content in PEP loaded electrospun fiber mats was analyzed by determining the menthol content by means of gas chromatography-mass spectrometry (GC-MS) using a GC 7890A and MSD 5975C (Agilent Technologies, Waldbronn, Germany) equipped with a DB-FFAP capillary column (30 m × 0.25 mm, film thickness 0.25 μm; Agilent Technologies, Santa Clara, CA). Firstly, the calibration curve was prepared from pure undiluted menthol at various concentrations (between 0.623 and 107 μg/ml) then, the solutions were injected into the GC-MS system. The concentration of menthol was calculated using the peak area (concentration = 916066 x peak area, *R*^2^ = 0.9959). After obtaining the menthol calibration curve, the electrospun fiber mats (~10 mg) were submerged in DCM (20 ml) for 2 h. Subsequently, the volatile fraction of PEP extraction was isolated by means of the solvent-assisted flavor evaporation technique (SAFE) (Engel et al., 1999). The volatile fraction in DCM was dried over sodium sulfate and then was filtered. The final volume was reduced to 100 μl using Vigreux- and microdistillation at 50°C (Bemelmans, 1979) Then, 1 μl of each sample was taken for the GC-MS measurements. The oven temperature was programmed at 40°C for 2 min and was then heated up at 8°C/min to 240°C and held for 5 min. Helium was used as carrier gas with a flow rate of 1 ml/min. Mass spectra were recorded with selected ion monitoring(SIM) mode (m/z ratio 71).

Afterwards, the percentage of encapsulation efficiency was calculated as follows (2):

(2) $$\begin{array}{l} Encapsulation\;Efficiency\;\left(EE\right)\left(\%\right)=\frac{m_{a}}{m_{t}}\;x\;100 \end{array}$$

Where, m_a_ and m_t_ are the actual and theoretical amounts of PEP in the electrospun fiber mats, respectively.

### Antibacterial Assay

The antibacterial activity of PCL and PCLPEP electrospun fiber mats was determined on *S. aureus* (Gram-positive) and *E. coli* (Gram-negative) bacteria. Firstly, the bacteria strains were incubated in lysogeny broth medium at 37°C for 24 h, and the optical density (OD) of the bacteria population was calibrated (600 nm, Thermo Scientific™ GENESYS 30™, Germany) to reach the value of 0.015, according to turbidity measurements of bacterial cultures. To assess the antibacterial activity of electrospun fiber mats, the samples were weighted (30 mg) and were sterilized by UV irradiation for 30 min prior to the experiment. Then, the samples were placed into a tube with 20 μl bacteria suspension, which was measured with the OD, in 2 ml broth medium. The samples were incubated at 37°C for various times (3, 6, 24, and 48 h). The OD value of the samples was measured at 600 nm after each incubation time interval, and relative bacterial viability was calculated as follows:

(3) $$\begin{array}{l} Relative\;bacterial\;viability\;\left(\%\right)=\;\frac{sample_{OD}}{control_{OD}}x100 \end{array}$$

The lysogeny broth medium and bacterial cell suspension in lysogeny broth medium were used as blank and control. Three parallel experiments were performed for each sample.

### WST-8 Cell Viability Assay

The cell viability of NHDF cells was analyzed on the PCL, PCLPEP1.5, PCLPEP3, and PCLPEP6 fiber mats. Firstly, NHDF cells were cultured in DMEM supplemented with 10% (FBS) and 1% penicillin/streptomycin solutions in 75 cm^2^ cell culture flasks (Nunc, Denmark). Counted cells were seeded into 24-well plates at a density of 50,000 cell/well and incubated at 37°C in a humidified incubator with 5% CO_2_ for 24 h. Prior to cell culture experiment, the nanofiber mats were fixed on CellCrownTM 24 inserts (ScaffdexOy, Tampere, Finland) and sterilized by UV light irradiation for 30 min. After 24 h incubation, the samples were immersed in a 24-well plate without touching the cell and incubated for further 48 h. After culturing the cells for 48 h, the viability of the cells was analyzed by a WST-8 cell counting assay kit (Sigma Aldrich) (with 5% WST-8 reagent in DMEM for a period of 2 h at 37°C). Finally, the absorbance of the obtained dye was measured at 450 nm using a spectrophotometric plate reader (PHOmo, anthos Mikrosysteme GmbH, Germany). The percentage of cell viability was calculated as follows:

(4) $$\begin{array}{l} Cell\;viability\left(\%\right)=\;\frac{Ab.\;of\;control-Ab.\;of\;test\;sample}{Ab.\;of\;control-Ab.\;of\;blank}x100 \end{array}$$

The absorbance of cell plus culture medium and WST-8 reagent were used as a control and blank. All samples were measured six times.

### Statistical Analysis

The statistical analysis of the data was carried out using one-way analysis of variance (ANOVA) with Origin (OriginLab, Northampton, MA, USA). To evaluate the statistically significant difference between groups, Bonferroni's test was applied, and *p* < 0.05 was considered to be significant.

## Results

### Surface Morphology and Diameter Distribution of Electropsun Fibers

**Figure 2:**
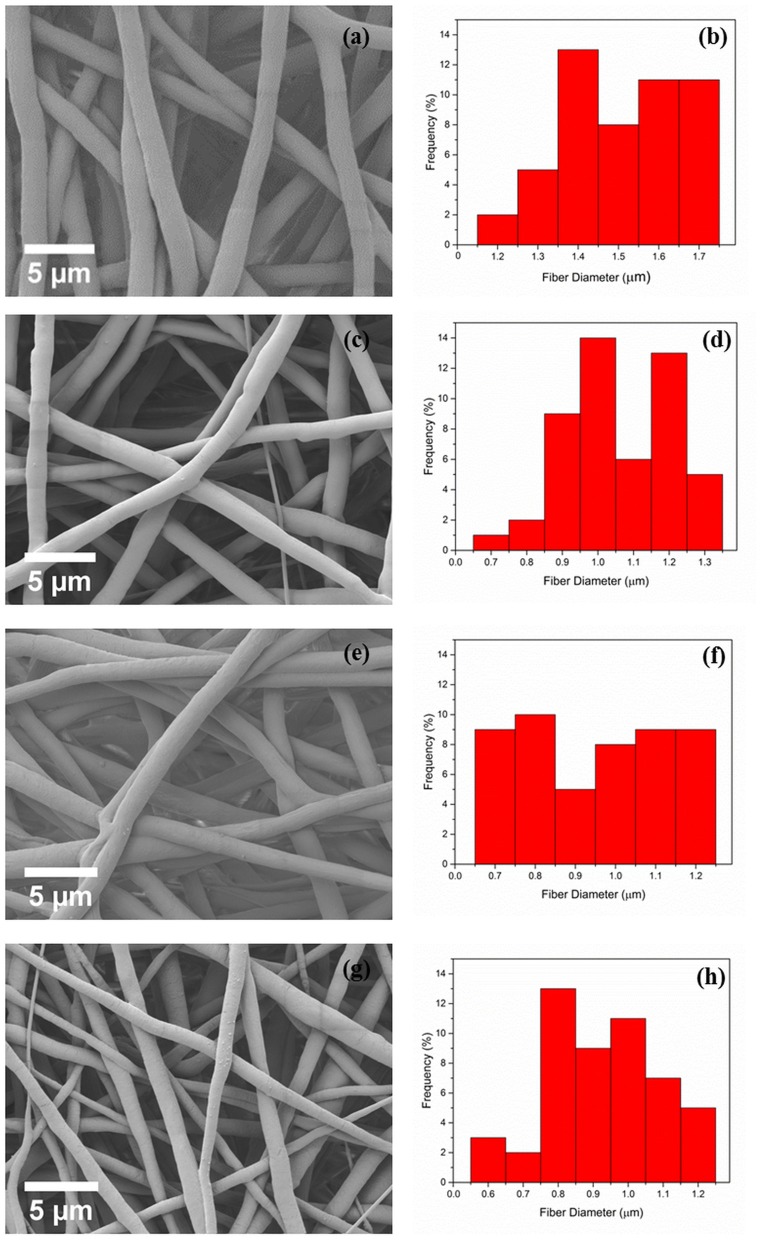
The morphology and fiber diameter distribution of PCL **(a,b)**, PCLPEP1.5 **(c,d)**, PCLPEP3 **(e,f)**, and PCLPEP6 **(g,h)** electrospun fiber mats, respectively.

Electrospun PCL and PCL fibers loaded with various amounts of PEP [1.5, 3, and 6% (v/v), namely PCLPEP1.5, PCLPEP3, and PCLPEP6] were fabricated by electrospinning method. Electrospinning processing parameters were optimized at a flow rate of 0.4 ml/h, the voltage of 15 kV and distance of 11 cm to reduce the number of beads on the fiber and fabricate homogenous, smooth fibers. All prepared mats were coded as shown in Table 1. Figure 2 illustrates that the morphology of the electrospun fiber mats was smooth, uniform, and bead-free. This result indicates no PEP aggregation on the fiber surface and that the incorporation of PEP in the PCL solution did not affect the fiber morphology. Additionally, the average fiber diameters of PCL, PCLPEP1.5, PCLPEP3, and PCLPEP6 fiber mats were 1.6 ± 0.2, 1.1 ± 0.2, 1.0 ± 0.2, and 0.9 ± 0.2 μm, respectively (Table 1). The distribution of fiber diameters indicates that the addition of PEP led to a slight decrease in fiber diameter as compared to neat PCL (Figure 1). However, increasing PEP concentration from 1.5 to 6% (v/v) did not significantly affect the average fiber diameter.

**Table 1:** Sample composition, sample label, average fiber diameter, and loading efficiency of electrospun fiber mats.

**Sample code**	**PCL (w/v %)**	**PEP (v/v %)**	**Average fiber diameter (μm)**	**Encapsulation efficiency (EE) (%)**
PCL	20	–	1.6 ± 0.1	–
PCLPEP1.5	20	1.5	1.1 ± 0.2	36 ± 14
PCLPEP3	20	3	1.0 ± 0.2	39 ± 10
PCLPEP6	20	6	1.0 ± 0.2	43 ± 7

### Raman Spectroscopy Analysis

**Figure 3:**
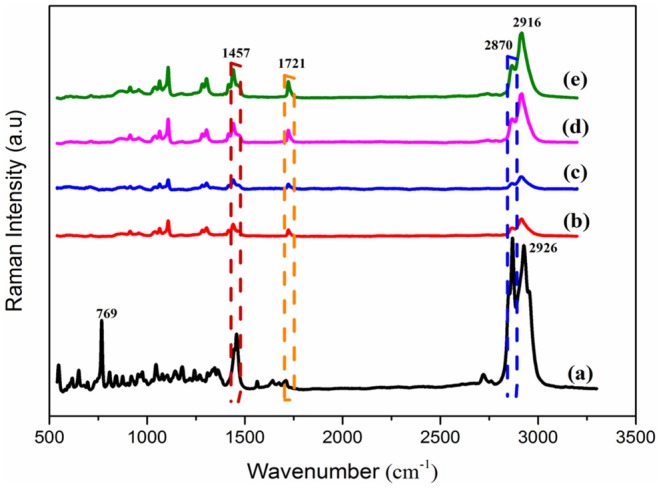
The Raman spectra of pure PEP **(a)**, PCL **(b)**, PCLPEP1.5 **(c)**, PCLPEP3 **(d)**, and PCLPEP6 **(e)** electrospun fiber mats.

The spectra of the Raman spectroscopy analysis of PEP and electrospun fiber mats are shown in Figure 3. As was reported in previous studies, menthol, menthone, and 1,8-cineole are the main components of PEP (Reverchon et al., 1994; Rohloff, 1999). Therefore, it was important to investigate the presence of such compounds in the fibers. The Raman spectrum of PEP exhibits peaks at 2870, 1457, and 769 cm^−1^, as illustrated in Figure 3a. The band at 2870 cm^−1^ is assigned to terpenoid vibrations of the PEP (Schulz et al., 2004). The other strong peaks at 1457 and 769 cm^−1^ correspond to CH_3_/CH_2_ bending of menthol and the ring deformation mode of menthol, respectively (Rösch et al., 2002; Jentzsch et al., 2015). As presented in Figure 3b, the typical peaks of PCL at 2916 and 2864 cm^−1^ can be related to the asymmetric and symmetric vibrations of the CH_2_ group. Further, the band for C=O vibration of the ester group was observed at 1721 cm^−1^ (Wesełucha-Birczyńska et al., 2015). These PCL peaks are evident in PCLPEP1.5, PCLPEP3, and PCLPEP6 electrospun fiber mats, whereas the bands of PEP only appeared in PCLPEP3 and PCLPEP6 (Figures 3c–e). Likewise, the typical band of PEP at 769 cm^−1^ did not appear in the Raman spectrum of PCLPEP1.5, PCLPEP3, and PCLPEP6. This could be attributed by the overlap with the PCL peaks.

### Wettability

**Figure 4:**
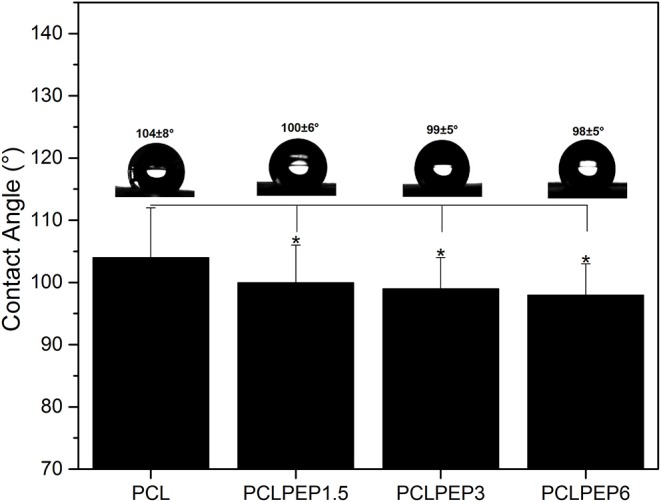
Water contact angle of PCL, PCLPEP1.5, PCLPEP3, and PCLPEP6 electrospun fiber mats (*n* = 3, sample in triplicate,*p < 0.05).

Figure 4 shows the contact angle values of PCL, PCLPEP1.5, PCLPEP3, and PCLPEP6 electrospun fiber mats. The average contact angle of PCL electrospun fiber mats was 104 ± 8°, whereas the PEP addition slightly decreased the contact angle to 98 ± 5°. However, no significant differences between PEP loaded PCL electrospun fiber mats, PCLPEP1.5 (100 ± 6°), PCLPEP3 (99 ± 5°), and PCLPEP6 (98 ± 5°) were found.

### *In vitro* Degradation

**Figure 5:**
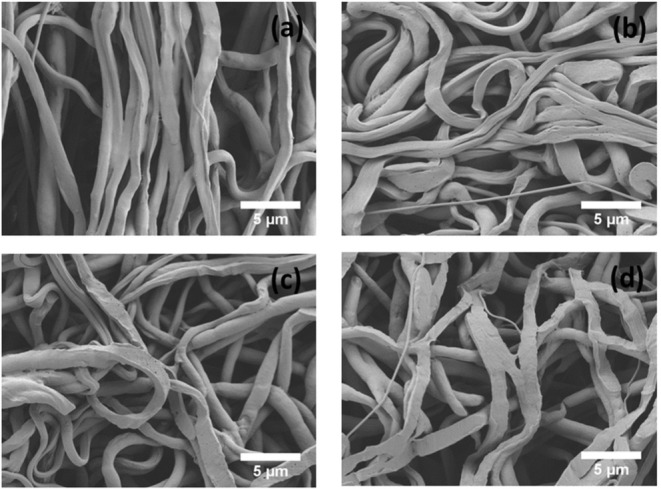
SEM images of PCL **(a)**, PCLPEP1.5 **(b)**, PCLPEP3 **(c)**, and PCLPEP6 **(d)** electrospun fiber mats after 14 days incubation in PBS at 37°C.

**Figure 6:**
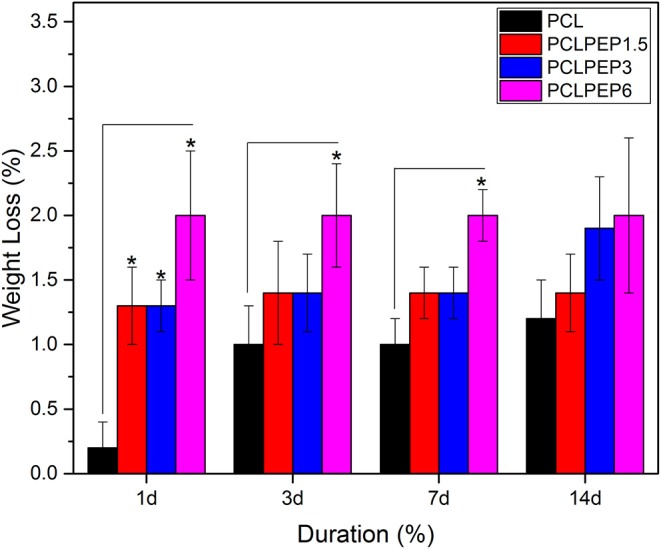
Weight loss percentage of PCL, PCPPEP1.5, PCLPEP3, and PCLPEP6 electrospun fiber mats incubated in PBS at 37°C for 1, 3, 7, and 14 days (*n* = 3, sample in triplicate,*p < 0.05).

An *in vitro* degradation study of electrospun fiber mats was conducted to evaluate the effect of PEP addition on PCL degradation in PBS. The morphology of electrospun PCL, PCLPEP1.5, PCLPEP3, and PCLPEP6 fiber mats after immersion in PBS for 14 days is illustrated in Figure 5. SEM images revealed that the fiber morphology was slightly degraded compared to the original structure. However, the overall fibrous morphology of samples mostly remained during the degradation study. Figure 6 shows the weight loss percentage of electrospun PCL, PCLPEP1.5, PCLPEP3, and PCLPEP6 fiber mats. The results demonstrated that the addition of PEP slightly increased the degradation rate compared to PCL electrospun fiber mats on 1d. However, there were no statistical changes among PCLPEP1.5, PCLPEP3, and PCLPEP6.

### Peppermint Oil Content in PCL Electrospun Fiber Mats

**Figure 7:**
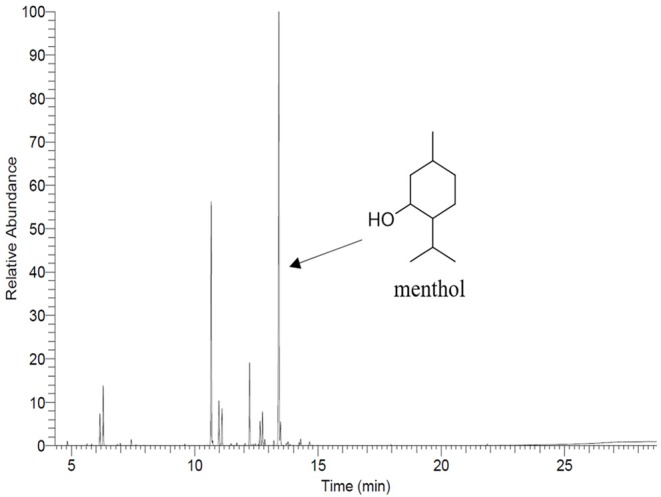
Gas chromatography profile of menthol showing it as a main component of PEP.

The main compound of PEP, menthol, was identified by GC using retention index and comparison with standard menthol (Figure 7). The actual amount of menthol in the pure PEP was 297 mg/ml. Besides, the encapsulation efficiency of PEP in PCL fiber mats was investigated by GC-MS spectroscopy. Table 1 shows the influence of the different concentrations of PEP on the percentage of encapsulation efficiency. The PEP encapsulation efficiency increased from 36 ± 14 to 43 ± 7% at PEP concentrations of 1.5 to 6 (v/v %). The results indicate that increasing PEP concentration has led to increased encapsulation efficiency.

### Antibacterial Assay

**Figure 8:**
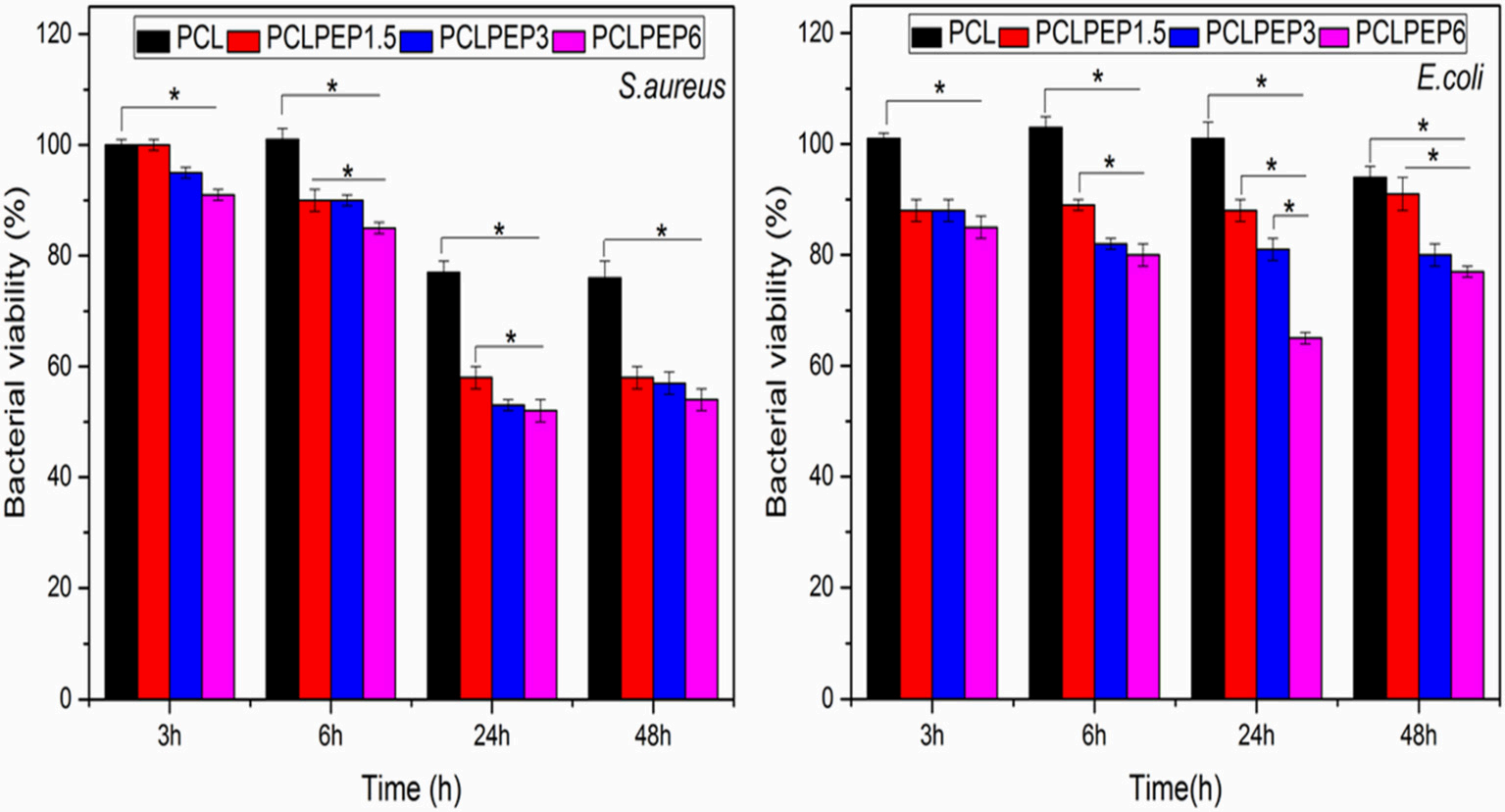
Relative bacteria viability of *S. aureus* (Gram-pozitive) and *E. coli* (Gram-negative) on the different type of electrospun fiber mats (PCL, PCLPEP1.5, PCLPEP3, and PCLPEP6) after 3, 6, 24, and 48 h incubation (*n* = 3, sample in triplicate,*p < 0.05).

The antibacterial activity of PCL, PCLPEP1.5, PCLPEP3, and PCLPEP6 electrospun fiber mats was tested with *Staphylococcus aureus* as gram-positive bacteria and *Escherichia coli* as gram-negative bacteria, separately. As presented in Figure 8, the relative bacterial viability of samples was investigated at 3, 6, 24, and 48 h. During the 48 h incubation, PCLPEP1.5, PCLPEP3, and PCLPEP6 exhibited decreased bacterial viability compared to PCL electrospun fiber mats. Additionally, the antibacterial activity was enhanced with an increase of PEP concentration in PCLPEP1.5, PCLPEP3, and PCLPEP6. The PCLPEP6 composition showed the lowest bacterial viability (*S. aureus*; 50 ± 3% and *E. coli*; 70 ± 2%) at 24 h incubation.

### WST-8 (Cell Viability) Assay

**Figure 9:**
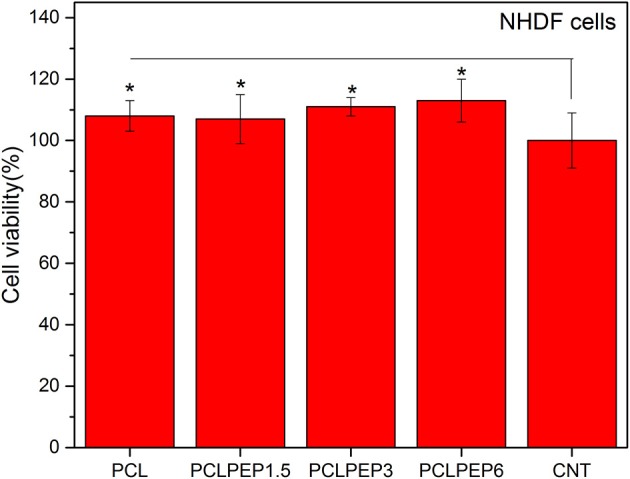
The cell viability of PCL, PCLPEP1.5, PCLPEP3, and PCLPEP6 electrospun mats in comparison with control (CNT) (*n* = 6,*p < 0.05).

The viability of NHDF cells was investigated to assess the biocompatibility of PCL, PCLPEP1.5, PCLPEP3, and PCLPEP6 fiber mats after 48 h of incubation, as shown in Figure 9. The results demonstrated that the cell viability of all fiber mats was increased compared to the control. However, there was no significant difference between each other. Our results thus indicated that PEP loaded PCL electrospun fiber mats exerted no toxic effects on NHDF cells.

## Discussion

EOs for antibiotic-free wound healing applications represent an emerging area of interest, which exploits the antibacterial activity, anti-inflammatory and antioxidative properties of EOs. This study investigates the various concentration of PEP loaded into electrospun PCL fiber mats in term of physical properties and antibacterial activity. Very few studies have reported on the electrospun fiber system incorporated with peppermint oil for wound healing applications. For instance, Liakos et al. (2015) fabricated homogenous, and bead-free cellulose/peppermint oil electrospun composite fibers for wound dressing; our results resemble their findings. In a related study, Jaganathan et al. (2019) reported on smooth and uniform electrospun polyurethane (PU) fibers of 635 nm ± 105 nm diameter using an electrospinning method adding PEP and copper sulfate (CuSO_4_) for wound dressing applications.

Data obtained in this study indicated that the average fiber diameter slightly decreased with the addition of PEP from 1.6 ± 0.2 to 0.9 ± 0.2 μm (Table 1). This could be due to the plasticizer effect of the essential oil (Mori et al., 2015). In this regard, PEP might be acting as a plasticizer, altering the order between single polymer chains and resulting in the decrease of solution viscosity. Further, the addition of PEP may increase the solution conductivity, which decreases the fiber diameter. Many studies have investigated that various electrospinning parameters such as polymer concentration, solution viscosity, flow rate of the polymer solution and solution conductivity affect the fiber morphology and fiber diameter and the solution of EO is expected to have an effect on these (Sill and von Recum, 2008; Bhardwaj and Kundu, 2010). According to Rieger et al. (2016), cinnamaldehyde (CIN) incorporation in chitosan/poly (ethylene oxide) nanofiber mats strongly decreased the solution viscosity (from 2.5 to 0.8 Pa s). In another study, Mani et al. (2018) reported that polyurethane composites containing neem oil showed a smaller fiber diameter due to the increase in the conductivity of the polymer solution. Similarly, Uslu et al. (2010) fabricated hybrid nanofibers based on poly (vinyl alcohol)/poly (vinyl pyrrolidone)/poly (ethylene glycol) incorporated with aloe vera. It was found that hybrid nanofibers exhibited a decreased diameter with increased electrical conductivity. The fact that PEP addition to PCL had no significant effect on PCL fiber morphology is important for applications, taking into account the need of developing a robust method for the reproducible production of fiber mats.

Based on the findings in this study, the addition of PEP slightly decreased the average contact angle (Figure 4). This result might be due to the chemical structure of PEP that includes an oxygenated group, which can interact with the H_2_O molecule. In a similar study, Jin et al. (2013) fabricated PCL nanofibers incorporated with four different essential oil extracts, namely *Indigofera aspalathoides* (IA), *Azadirachta indica* (AI), *Memecylon edule* (ME), and *Myristica andamanica* (MA). Their results indicated that the addition of essential oil decreased the contact angle value compare to pure PCL (Jin et al., 2013), which is in agreement with our results. In another study, findings of Agnes Mary and Giri Dev (2015) indicated that PCL electrospun matrices comprising aloe vera (AV) had lower contact angle values when the AV concentration increased.

In general, the degradation rate of biopolymers depends on crucial factors such as crystallinity, hydrophilicity, molecular weight, morphological structure, pH, and temperature (Göpferich, 1996). As illustrated in Figure 6, the weight loss percentage of electrospun fiber mats slightly increased with the addition of PEP. This result might be attributed to the reduction of intermolecular forces between the PCL chains due to the addition of PEP. Furthermore, the wettability results (Figure 4) indicated that addition of PEP slightly reduced the contact angle value, which could also affect the degradation rates. In a similar study, Agnes Mary and Giri Dev (2015) found that the degradation of PCL fibers containing Aloe vera (AV) degraded faster due to the increase of the hydrophilicity of fibers (Agnes Mary and Giri Dev, 2015). In another study, Sadri et al. (2015) reported that the amount of green tea extract increased the degradation of chitosan/polyethylene oxide polymeric nanofibers (Sadri et al., 2015).

On the other hand, the encapsulation efficiency of PEP in PCL electrospun fiber mats was investigated. Table 1 shows the effects of the various initial amounts of PEP on encapsulation efficiency. Increasing the initial PEP amount resulted in an overall increase in the amount of PEP encapsulation in the electrospun fiber mats. Similarly, Tampau et al. (2017) investigated the influence of different polymer and carvacrol concentrations on the encapsulation efficiency and found that the encapsulation efficiency increased from 15 to 75% at 5 to 15 wt% carvacrol ratio. Balasubramanian and Kodam (2014) fabricated different concentrations of lavender essential oil encapsulated in polyacrylonitrile (PAN) nanofibers for antibacterial applications. It was revealed that the encapsulation efficiency of fibers ranged from 25.8 to 32.4%. Moreover, the encapsulation efficiency increased with increasing lavender oil concentration in the fibers. In another study, Tavassoli-Kafrani et al. (2018) reported that the encapsulation efficiency of gelatin nanofibers incorporating at different ratios of orange EO increased as the concentration of orange oil increased.

The antibacterial activity of the PEP loaded PCL electrospun fiber mats was tested with *S. aureus* and *E. coli* bacteria. PEP is known to be a potent antimicrobial against a variety of microorganisms by interacting with their cell membrane (McKay and Blumberg, 2006; Kligler and Chaudhary, 2007). Our results indicated that the addition of PEP reduced the bacteria viability for both bacteria strains, as shown in Figure 8. Recently, Li et al. (2018) reported that eugenol loaded PCL/gelatin(Gel) electrospun membranes exhibited increasing antibacterial properties with increasing the concentration of eugenol. Moreover, the present samples exhibited better bacterial inhibition toward *S. aureus* than *E. coli*. Gram-negative bacteria such as *E. coli* consist of a double membrane and the outer membrane has a lipopolysaccharide layer which prevents penetration of antibacterial compounds (Hammer et al., 1999; Burt and Reinders, 2003; Burt, 2004). Therefore, PEP loaded electrospun fiber mats could be less effective in their action against *E. coli*. Wen et al. (2016) found similar results for cinnamon oil incorporated PLA (polylactic acid) electrospun nanofibrous film.

Data presented here indicated that there were no significant differences (*p* < 0.05) in the cell viability of the electrospun mats in comparison with the control (Figure 9). The *in vitro* cell viability results demonstrated that PCL, PCLPEP1.5, PCLPEP3, and PCLPEP6 electrospun fiber mats exerted no cytotoxic effect on NHDF cells. In agreement with our results, Tang et al. (2019) reported that peppermint and chamomile loaded gelatin nanofibers did not change NIH-3T3 fibroblast cell viability. In another study, Hajiali et al. (2016) investigated the antibacterial activity and biocompatibility of alginate-lavender nanofibers. The cell viability of lavender incorporated alginate nanofibers was 91%. It was stated that the addition of lavender oil did not affect cell viability of human foreskin fibroblast (HFF-1) cells compared to the control group.

Given the large number of studies emerging on the use of EOs in different polymers, the relative antibacterial effectiveness of electrospun fibers incorporating different EOs should be investigated.

## Conclusion

In this study, electrospun PCL fibers at different concentrations of PEP [1.5, 3, and 6 (v/v)%] were developed and characterized. The fibers were smooth and uniform under optimum electrospinning parameters. The morphological analysis showed that the average fiber diameter was reduced from 1.6 ± 0.1 to 1.0 ± 0.2 μm when loading PEP. The presence of PEP was confirmed by Raman spectroscopy and GC-MS analysis. Furthermore, the antibacterial activity of PCLPEP electrospun fiber mats was evaluated against both bacteria strains (*S. aureus* and *E. coli*). Our results showed that bacterial inhibition was effective on PEP loaded PCL electrospun fiber mats, especially PCLPEP6. Moreover, the cell viability and antibacterial assay results indicated that the addition of PEP did not induce cytotoxicity, although it led to high antibacterial activity. Consequently, PEP loaded PCL electrospun fiber mats have potential for applications in antibiotic-free bacterial infection treatment as wound healing materials.

## Data Availability Statement

The raw data supporting the conclusions of this manuscript will be made available by the authors, without undue reservation, to any qualified researcher.

## Author Contributions

IU and ARB conceived the presented idea. IU fabricated the samples and performed the experiments, analyzed the data, and wrote the manuscript. BS and IU performed the Gas Chromatography-Mass Spectroscopy analysis and collected the data. WG helped carry out the antibacterial activity assay. GF assisted with Raman Spectroscopy measurements. ARB was involved in the planning and supervision of the project. All authors discussed the results, provided critical feedback and contributed to the final manuscript.

### Conflict of Interest

The authors declare that the research was conducted in the absence of any commercial or financial relationships that could be construed as a potential conflict of interest.
